# Overcoming Barriers to Applying Systems Thinking Mental Models in Policy-Making Comment on "What Can Policy-Makers Get Out of Systems Thinking? Policy Partners’ Experiences of a Systems-Focused Research Collaboration in Preventive Health"

**DOI:** 10.34172/ijhpm.2020.50

**Published:** 2020-04-08

**Authors:** Sobia Khan

**Affiliations:** ^1^The Center for Implementation, Toronto, ON, Canada.; ^2^University of Toronto, Toronto, ON, Canada.

**Keywords:** Systems Thinking, Complexity, Policy-Making

## Abstract

Systems thinking provides the health system with important theories, models and approaches to understanding and assessing complexity. However, the utility and application of systems thinking for solution-generation and decision-making is uncertain at best, particularly amongst health policy-makers. This commentary aims to elaborate on key themes discussed by Haynes and colleagues in their study exploring policy-makers’ perceptions of an Australian researcher-policy-maker partnership focused on applications of systems thinking. Findings suggest that policy-makers perceive systems thinking as too theoretical and not actionable, and that the value of systems thinking can be gleaned from greater involvement of policy-makers in research (ie, through co-production). This commentary focuses on the idea that systems thinking is a mental model that, contrary to researchers’ beliefs, may be closely aligned with policy-makers’ existing worldviews, which can enhance adoption of this mental model. However, wider application of systems thinking beyond research requires addressing multiple barriers faced by policy-makers related to their capability, opportunity and motivation to action their systems thinking mental models. To make systems thinking applicable to the policy sphere, multiple approaches are required that focus on capacity building, and a shift in shared mental models (or the ideas and institutions that govern policy-making).

The complexity of the health system is perplexing to those working within it and is a system characteristic that many have long grappled with. In an effort to understand systems, and particularly *complex* systems, systems thinking has gained traction as a set of theories and approaches that aim to make sense of complexity.^1^ Actors within a system perceive complexity when there is a high degree of uncertainty, with no clear potential solutions.^2^ For individuals tasked with decision-making within complex systems, this is the worst possible case. Uncertainty hinders rational decision-making^3^; compound this uncertainty with risk mitigation (which is a particular concern in health systems), and substantive rationality substantially decreases.

Systems thinking demands that we face uncertainty head on, despite the discomfort that may result in doing so.^4^ This is counter to how health system actors, including policy-makers, have traditionally been trained to deal with uncertainty – which is to apply methods we know work in simple systems for simple problems, and hope we can garner the same results in far more complex conditions. What we (ie, systems scientists and practitioners) are essentially aiming to stimulate is a shift to more appropriate and innovative tactics that address the challenges that ail us. Policy-makers in complex systems have a particularly tall order. Policy solutions must be outwardly simple to appeal to a broad range of stakeholders, despite the inherent complexity of process of arriving at policy options and the often quiet complexity of the options themselves. Moreover, policy action can set the stage for large scale change and system resilience, but can also pose the largest roadblocks for change and foster system brittleness.

So how can we support policy-makers in complex health systems? In their study, Haynes et al^5^ state that “people make sense of the world given what they know so, without a compelling rationale [to adopt systems thinking], we tend to hold on to established mental models and avoid the disruption of seeing the world in radical new ways.” This statement assumes that systems thinking is strongly divergent from the existing mental models that policy-makers hold about their world. I offer the view that policy-makers’ individual mental models are likely more aligned with systems thinking than assumed, and that it is the incapacity to apply those mental models that pose some of the biggest barriers to how systems thinking can be adopted and enacted. As such, we may require different approaches to support policy-makers in pursuit of improved complex systems solutions.

Mental models are defined as the cognitive processes we use to understand and produce expectations about our environment.^6^ The cognitive “infrastructure” we use to process this information is developed through experience, culture, social interaction, and knowledge acquisition, among other factors. Mental models are important because they can shape individual behaviour.^6^ When mental models are collective or shared, they govern social and political ideas and institutions.^3^ In order to be able to make decisions in an uncertain (complex) environment, policy-makers require mental models to help them understand the problem; they then identify potential solutions and understand the possible outcomes of those solutions in their decision-making processes.^6^

Policy-makers inherently know that they work in complex environments because policy-making is not linear. The act of weighing evidence and interests, and of generating and balancing multiple possible options, is a reflection of the complexity that plagues the policy landscape. As such, fostering a systems thinking mental model is not necessarily vastly different from policy-makers’ existing worldviews. What systems thinking may offer is a set of theories and approaches that enable policy-makers to articulate what they have already been observing. Denzau and North eloquently stated that adopting complex mental models is “…a process by which we substitute a familiar complexity for one that we have found novel. The invisible hand result is now obvious and intuitive not because it is simple, but because we are trained to see it when it may be present or useful.”^3^ As such, it may be useful to position systems thinking not as a radical new mental model, but one that reframes policy-makers’ old familiar ideas of complexity while offering an improved approach to exploring problems.

Fostering a systems thinking mental model is the first step in stimulating the use of systems approaches among policy-makers. This is often the outcome of capacity building efforts in systems thinking. However, using systems thinking in action requires the capability, opportunity and motivation to do so.^7^ Policy-makers become capable to take action in a system when they are able to draw on their knowledge and skills to identifying solutions and potential outcomes of those solutions in ways that acknowledge and address complexity. This is where systems thinking is lacking. As described in Haynes and colleagues’ study, policy-makers see systems thinking as explanatory but not action-oriented.^5^ The “action” part of systems thinking is rooted in the hard methodologies that suit the needs of researchers. There has been little advancement on how to adapt and teach the soft methodologies of systems thinking for the purposes of policy-making. When faced with uncertainty, a policy-maker may hold a systems thinking mental model, but will rely on their prior knowledge and skills related to managing uncertainty (ie, simple solutions applied to complex problems) in the absence of a viable alternative.^3^ Therefore, building capabilities to take action in complex environments is crucial.

Moreover, there may be a lack of opportunity to be able to apply systems thinking in a policy context. While individual mental models can be shifted to align more closely with systems thinking, our collective mental models – the *ideas* and *institutions* that are imperative to action in the policy sphere^8^ – may experience a slower shift in this direction. Ideas at a socio-political level are the shared mental models that we internalize collectively – for example “patient centeredness,” “evidence-informed policy,” and “systems thinking.” Institutions are the structural manifestations of these shared mental models, such as government structures and policy legacies.^3^ Individuals who subscribe to a systems thinking mental model may experience friction when attempting to action their worldview if ideas remain the status quo, and institutions continue to reflect and perpetuate the status quo. One example is the concept of distributed power, which is difficult to enact when organizations and systems still subscribe to traditional ideas about leadership and maintain top-down hierarchical structures. Thus, there is a lack of opportunity to exercise systems thinking when old norms and structures still reign.

Finally, when the capability and opportunity to apply systems thinking are inadequate, many policy-makers may not feel motivated to adopt and apply and systems thinking mental model. Motivation is born from the idea that this shift will lead to tangible beneficial outcomes.^3^ As noted previously, despite the complexity of the policy solutions themselves, there is often an expectation that the outward appearance of the solution is simple and straightforward. Therefore, the external legitimacy and acceptance of using a systems thinking approach may be questioned if the end result cannot be distilled into clear, actionable components. Even if individual policy-makers believe that systems thinking is the correct worldview, most cannot be motivated to adopt it if they cannot practice it, unless they have the deep intrinsic motivation to do so.

In order to address these challenges and truly support policy-makers to embrace systems thinking, a few key actions may be required. First, capacity building efforts ideally should not be researcher-led, but can be collaboratively designed and delivered by both policy-makers and researchers. This is because systems thinking mental models may differ between subgroups (given that subgroups have different education, experiences, and applications of systems thinking) even though the entirety of the group subscribes to a systems thinking worldview. There is a lack of fit when researchers try to impose their ideas of systems thinking onto people who do not conduct research, and vice versa. A better solution is to develop curricula premised on shared sense-making^9^ about systems thinking (ie, creating a “common sense” about systems thinking across diverse subgroups) and foster learning through both inductive and deductive practices.^10^ Meaning, people learn when they see familiarity in the mental model that is presented, and when they can both draw from their own experiences to inform rules, and apply rules they have learned to their experiences. Moreover, those of us working in the system thinking sphere may need to continue building tools, approaches and resources that enhance research-informed practice (ie, taking what we know from research such as soft and hard systems methodologies, and adapting these for policy-makers), and that can be synthesized through practice-informed research^11^ (ie, understanding and evaluating what innovative practices are being performed amongst policy-makers to address system complexity).

Second, we can aim to bolster ideas related to systems thinking and alter institutions to enable the opportunity for individuals to apply it. Ideas can be perpetuated through discourse and championing of systems thinking approaches. This discourse should occur between policy-makers and include other relevant actors such as researchers, rather than having discourse directed to policy-makers. Institutions can start making space for systems thinking in different ways. For example, Uhl-Bien et al describe the development of “adaptive spaces” within organizations to work both against and within traditional leadership structures.^12^ The adaptive spaces are meant to allow for rapid generation and testing of solutions, and rely on a flattening of power structures within that space to protect innovation. These ideas are ultimately meant to be siphoned by the organization for widespread implementation. Another example is recognition of systems thinking within provincial government bodies in Saskatchewan, Canada, and having this drive changes in provincial governance structures to foster better system connectivity.^13^

The approaches described here leverage praxis, but also go beyond praxis in an attempt to truly foster change. Mental models can be at odds with systemic barriers that oppose the application of those models. However, starting with a shift in mental models and the acquisition of a knowledge base about systems thinking is a good starting point. A multi-pronged approach to support policy-makers can be implemented alongside capacity building efforts to truly stimulate the capability, opportunity and motivation to adopt and apply systems thinking.

## Ethical issues

Not applicable.

## Competing interests

Author declares that she has no competing interests.

## Author’s contribution

SK is the single author of the paper.
